# Myosin VI in skeletal muscle: its localization in the sarcoplasmic reticulum, neuromuscular junction and muscle nuclei

**DOI:** 10.1007/s00418-012-1070-9

**Published:** 2012-12-30

**Authors:** Justyna Karolczak, Magdalena Sobczak, Łukasz Majewski, Marine Yeghiazaryan, Anna Jakubiec-Puka, Elisabeth Ehler, Urszula Sławińska, Grzegorz M. Wilczyński, Maria Jolanta Rędowicz

**Affiliations:** 1 Department of Biochemistry, Nencki Institute of Experimental Biology, 3 Pasteur St., 02-093 Warsaw, Poland; 2 Department of Neurophysiology, Nencki Institute of Experimental Biology, 3 Pasteur St., 02-093 Warsaw, Poland; 3 Cardiovascular Division, King’s College London, 125 Coldharbour Lane, London, SE5 9NU UK

**Keywords:** Cytoskeleton, Muscle fiber, Myosin VI, Neuromuscular junction, Nucleus, Sarcoplasmic reticulum

## Abstract

Myosin VI (MVI) is a unique unconventional motor moving backwards on actin filaments. In non-muscle cells, it is involved in cell migration, endocytosis and intracellular trafficking, actin cytoskeleton dynamics, and possibly in gene transcription. An important role for MVI in striated muscle functioning was suggested in a report showing that a point mutation (H236R) within the MVI gene was associated with cardiomyopathy (Mohiddin et al., J Med Genet 41:309–314, 2004). Here, we have addressed MVI function in striated muscle by examining its expression and distribution in rat hindlimb skeletal muscle. We found that MVI was present predominantly at the muscle fiber periphery, and it was also localized within muscle nuclei. Analysis of both the hindlimb and cardiac muscle longitudinal sections revealed ~3 μm striation pattern, corresponding to the sarcoplasmic reticulum. Moreover, MVI was detected in the sarcoplasmic reticulum fractions isolated from skeletal and cardiac muscle. The protein also localized to the postsynaptic region of the neuromuscular junction. In denervated muscle, the defined MVI distribution pattern was abolished and accompanied by significant increase in its amount in the muscle fibers. In addition, we have identified several novel potential MVI-binding partners, which seem to aid our observations that in striated muscle MVI could be involved in postsynaptic trafficking as well as in maintenance of and/or transport within the sarcoplasmic reticulum and non-sarcomeric cytoskeleton.

**Electronic supplementary material:**

The online version of this article (doi:10.1007/s00418-012-1070-9) contains supplementary material, which is available to authorized users.

## Introduction

Myosins form a structurally and functionally diverse superfamily of actin-based molecular motors that consists of more than 35 distinct families (Odronitz and Kollmar 2007). The family classification is based on the diversity of amino acid sequences at the N-terminal motor domain (containing the ATP- and actin-binding sites), the region responsible for myosin motor activity.

Several unconventional myosins have been identified in striated muscle and myogenic cells, namely myosin I isoforms (IA, I B and IC), myosin VA, myosin VI, myosin VIIA, myosins IXa and IXB as well as myosin XVIIIB (reviewed by Redowicz 2007). However, very little is known about their roles in muscle tissue and the non-sarcomeric actin cytoskeleton (Kee et al. 2009). The first observation on the possible involvement of myosin VI (MVI) in muscle function was made by Mohiddin et al. (2004) who showed that in humans a point mutation within the MVI gene was associated not only with deafness, but also with mild symptoms of cardiac hypertrophy. Notably, all mutations identified so far within vertebrate MVI genes have been associated with deafness.

The MVI heavy chain (~140 kDa) is aligned in a domain pattern characteristic for all the myosins described so far: the motor domain, a neck (with one classical IQ motif, to which calmodulin binds) and a tail domain. The C-terminal part of the tail forms a globular domain, which is essential for cargo binding and/or interaction with binding partners as well as with PIP_2_-containing liposomes (Sweeney and Houdusse 2007, 2010; Chibalina et al. 2009). Due to the presence of two inserts (small and large ones) within the tail domain, four splice variants of MVI could be formed in mammalian cells (Au et al. 2007; Aschenbrenner et al. 2003). The authors postulated that the inserts could determine its subcellular localization and function.

MVI seems to be a processive motor walking on actin filaments; however, unlike other myosins characterized so far, it moves toward the minus (pointed) end of actin filaments (Wells et al. 1999). This unique feature implies that it may play different roles than other myosins engaged in the same processes. Increasing evidence indicates that MVI is engaged in a variety of cellular functions including endocytosis and intracellular transport of vesicles and organelles, cell migration, maintenance of Golgi apparatus, actin cytoskeleton organization, and possibly gene transcription (Sweeney and Houdusse 2007, 2010; Chibalina et al. 2009; Vreugde et al. 2006; Jung et al. 2006).

It has been widely accepted that MVI fulfills its functions through interactions with actin and a set of its interaction partners bound to the MVI C-terminal globular tail domain (also termed the cargo domain) in tissue- and cell type-specific manners. Two regions within the cargo domain have been found to be involved in recognition of its partners: the positively charged RRL region and the hydrophobic WWY region (Chibalina et al. 2009). Also, a positively charged region of the MVI C-terminal globular tail was found to bind to PIP_2_-containing liposomes (Spudich et al. 2007), which could aid in the partner binding. Several MVI binding-partners have been identified so far in mammals: Disabled homolog 2 (Dab2), synapse-associated protein 97 (SAP97), GAIP-interacting protein COOH terminus (GIPC), lemur tyrosine kinase 2 (LMTK2), optineurin, phospholipase Cδ3, TRAF6-binding protein (T6BP), nuclear dot protein 52 (NDP52), translocated in liposarcoma (TLS) and dedicator of cytokinesis 7 (DOCK7) (Aschenbrenner et al. 2003; Chibalina et al. 2007; Majewski et al. 2012; Morris et al. 2002; Morriswood et al. 2007; Sakurai et al. 2011; Tamaki et al. 2009; Wu et al. 2002).

The involvement of MVI in neurotransmission has also been postulated since severe defects in hippocampal neurons were detected in Snell’s waltzer (SV) mice which lack MVI (Lewis et al. 2011; Osterweil et al. 2005; Yano et al. 2006). These defects might be responsible for the neurological symptoms observed in SV mice such as head tossing and hyperactivity (Redowicz 2002). It has also been shown that in *Drosophila*, MVI is present in the neuromuscular junction, thus suggesting its involvement in neuromuscular transmission (Kisiel et al. 2011). This and other observations described above (including the association of the MVI gene mutation with hypertrophic cardiomyopathy) further indicate that MVI could be engaged in striated muscle functioning.

To date, no further studies aimed at elucidation the role of MVI in striated muscle have been presented. Here, we present, for the first time, data addressing this issue by determining the distribution and expression pattern of MVI in the normal and denervated rat hindlimb skeletal muscle as well by identifying potential muscle proteins interacting with this unconventional myosin.

## Materials and methods

### Animals

Wistar 3-month-old rats were used in the studies. Murine hearts were excised from terminally anesthetized and heparinized C57B mice (body weight 20–25 g). Housing and surgical procedures were performed in compliance with the European Communities Council Directive of 24 November 1986 (86/609/EEC), and the protocol was approved by the First Warsaw Local Ethics Committee for Animal Experimentation, Poland. Efforts were made to minimize animal suffering and to use the number of animals absolutely necessary to produce reliable scientific data. Usually, muscles from at least four animals were used in a given experiment. The hindlimb muscles were also collected from four amyotrophic lateral sclerosis (ALS) rats with a knock-in of the human superoxide dismutase 1 gene (*SOD1*) with G93A mutation [a gift from Prof. Pawel Grieb from the Mossakowski Medical Research Center in Warsaw (Grieb 2004)]. Animals used for the analysis were in the terminal stage of the disease, characterized by significant weight loss and complete paralysis of both hindlimbs and at least one forelimb.

### Denervation procedure

Hindlimb muscles were denervated by cutting the sciatic nerve in anaesthetized (Equithesin 0.35 ml/100 g b.w.) female rats under sterile conditions as described by Jakubiec-Puka et al. (2008). Both nerve stumps were strongly ligated, and the proximal stump was implanted into the subcutaneous dorsal region. Two months after denervation, the animals were decapitated and hindlimb muscles (soleus, gastrocnemius medialis, GM and extensor digitorum longus, EDL) were isolated.

### Preparation of muscle homogenates

Isolated muscles from either control or denervated animals were homogenized in 10 volumes of ice-cold 20 mM K_2_HPO_4_/KH_2_PO_4_ buffer, pH 7.2 containing 1 mM phenylmethylsulfonyl fluoride (PMSF) supplemented with the complete protease inhibitor cocktail (Roche Diagnostics GmbH, Germany). Homogenates were next subjected to SDS polyacrylamide gel electrophoresis.

### Isolation of sarcoplasmic reticulum fractions

Fractions of sarcoplasmic reticulum (SR) vesicles of skeletal and cardiac muscle were prepared according to Wrzosek et al. (2012). To obtain the skeletal SR fraction, the white back muscle and the leg muscle (fast twitch muscle) were isolated from Wistar rats weighing approximately 250 g. To prepare the cardiac SR fraction, the ventricular tissue was excised from hearts also isolated from Wistar rats weighing between 250 and 350 g. The fractions after separation on 12 % SDS polyacrylamide gel (about 20 μg of protein of each sample was loaded) were subjected to western blot analysis with anti-MVI and anti-calreticulin antibodies.

### Analysis of myosin VI splice forms by RT-PCR

Total RNA was isolated from soleus and EDL muscles with the RNeasy Fibrous Tissue Mini Kit (Qiagen), followed by the synthesis of cDNA using the ReverseAid™ First Strand cDNA Synthesis Kit (Fermentas) with oligo-dT primers and subsequent RT-PCR analysis. Primers flanking the sequence of the small and large inserts of the rat myosin VI gene were designed and used to amplify the regions of interest [small insert: (forward: 5′-GATGAGGCACAGGGTGAC-3′ and reverse: 5′-GCGTATTTCCATTTACTGAGA-3′; large insert: forward 5′-GCTCCCAAGTCGGTTACT-3′ and reverse 5′-TTGTTCTGAGGGTCTTTGTA-3′)]. The housekeeping glyceraldehyde-3-phosphate dehydrogenase (GAPDH) gene was used as an internal control. PCR products were run on a 3 % agarose gel that, after staining with ethidium bromide, was photographed using G:Box from SynGene (Cambridge, UK) equipped with Gene Snap software.

### Antibodies and fluorescent markers

Rabbit polyclonal antibody directed against the amino-acid residues 1049–1054 of the porcine myosin VI heavy chain was purchased from Proteus (USA). The specificity of the antibody in the muscle tissue was confirmed by western blot analysis of hindlimb muscle homogenates as shown in the Online Resource 1. Goat polyclonal antibody against SERCA 1 pump was from Santa Cruz Biotechnology (USA), and the antibody against human calreticulin was a gift from Dr. Marek Michalak from the University of Alberta (Canada). The following monoclonal antibodies were also used: anti-GM130 from BD Biosciences (USA), anti-GAPDH (glyceraldehyde-3-phosphate dehydrogenase) from Merck Millipore (USA), anti-dystrophin and anti-SERCA 2 from Abcam (UK), anti-α-actinin from Santa Cruz Biotechnology (USA), and anti-synaptophysin from Dako (Denmark). ToPro3 (a dye staining chromatin) and Alexa488-conjugated bungarotoxin (staining muscle synapses) were purchased from Invitrogen (USA).

### Immunoblotting

Homogenates of control and denervated soleus and EDL muscles (10–20 μg of protein) or sarcoplasmic reticulum fractions of skeletal and cardiac muscles were separated using 10 or 12 % polyacrylamide SDS gels and then transferred to a nitrocellulose membrane. After the transfer, the membrane was blocked for 1 h at room temperature in tris-buffered saline (TBS) containing 5 % non-fat milk powder and 0.2 % Triton X-100 followed by 1-h incubation with appropriate dilutions of the antibodies against myosin VI, calreticulin and GAPDH (usually 1:200–1:500 dilutions were applied except of anti-GAPDH antibody, which was used at 1:5,000 dilution). The primary antibodies were detected using 1:10,000 dilutions of anti-rabbit and anti-mouse as well as 1:50,000 dilution of anti-goat antibodies conjugated with horse radish peroxidase; the reaction was developed using ECL according to the manufacturer’s instructions (Pierce, USA).

### Densitometric analysis

Developed blots were photographed using the G:Box system equipped with GeneSnap and GeneTools software. The content of MVI and GAPDH was estimated by densitometric analysis. Samples obtained from denervated muscles were compared with those obtained from control (intact) muscle, run in the same gel. A Student’s *t* test was used to evaluate the quantitative data.

### Immunolocalization studies

Fourteen micrometer thick transverse and longitudinal cryosections of soleus, EDL or GM muscles were fixed in 4 % formaldehyde (freshly prepared from paraformaldehyde) in phosphate-buffered saline pH 7.4 (PBS) for 30 min at room temperature, washed with PBS, blocked in 5 % normal goat serum and permeabilized with 0.3 % Triton X-100 in PBS for 30 min at room temperature. The same procedure was applied for longitudinal sections of cardiac ventricular muscle. Slides were then incubated with anti-MVI rabbit polyclonal antibody diluted 1:50 and next with sarcoplasmic reticulum markers: anti-calreticulin antibody diluted 1:50 or anti-SERCA1 (or anti-SERCA2) antibody diluted 1:50 (200 μg/ml IgG) overnight at 4 °C, washed with PBS, followed by incubation with 1 μg/ml Alexa Flour 488 or Alexa Fluor 546 conjugated goat anti-rabbit IgG or donkey anti-goat IgG (Molecular Probes, Invitrogen). Also immunostainings using antibodies against dystrophin to stain cytoplasmic side of sarcolemma (1:100), α-actinin to label Z-disk (1:50), GM130 to label the cis Golgi network (1:200) and synaptophysin to label the presynaptic domain of the neuromuscular junction (1:20) were performed. Nicotinic acetylcholine receptors were detected by 5 μg/ml α-bungarotoxin Alexa Fluor conjugates. The nuclei were stained with ToPro3 dye diluted 1:1,500 in PBS for 30 min at room temperature. To mount the slides, Vectashield mounting medium was used (Vector Labs, USA). Images were collected with the Leica TCS SP2 or SP5 confocal laser scanning microscopes equipped with a 63× HCX Oil CS UV 1.4 oil-immersion Plan Apochromat lens. An argon laser at 488 nm, a diode pumped solid-state laser 561 nm and a helium neon laser at 594 were used to excite Alexa 488, 555 and 546 fluorescence, respectively. Optical sections (1,024 × 1,024 pixels × 12 bits/pixel) were collected usually at 0.30 μm z-spacing. In double or triple immunostaining, special care was taken to control for any possible cross-talk of the detection systems. We carefully adjusted the spectral ranges of the detectors and always scanned the images sequentially. For negative controls, the primary antibody was omitted. A quantitative assessment of fluorophore co-localization in confocal optical sections was performed using Pearson’s correlation coefficient, which is a well defined and commonly accepted tool for describing the extent of overlap between image pairs (Manders et al. 1993). The value of this coefficient ranges from −1 to 1, with a value of −1 representing a total lack of overlap between pixels from the images, and a value of 1 indicating perfect image registration. Area of interest (AOI) within the scatter plot marked the colocalized areas and the resulted mask reflects overlapping regions of red and green channels.

### Purification of GST-fusion protein

The fusion protein composed of GST and MVI C-terminal globular tail domain (GST-MVI-GD), and GST alone were obtained as described by Majewski et al. 2012. Briefly, *E. coli* BL21(DE3)pLysS bacteria (Novagen, USA) transformed with the GST-MVI-GD or GST alone expression plasmids were grown in LB medium with 1 % glucose until OD_60_ = 0.6, and then protein expression was induced by 0.2 mM IPTG. After 2-h incubation at 25 °C, the bacterial cultures were harvested by centrifugation. Purification of the expressed proteins was performed on affinity chromatography Glutathione Sepharose 4B™ resin according to the manufacturer’s instruction (Amersham Biosciences, Sweden).

### GST pull-down

EDL muscle was homogenized in 10 volumes (w/v) of ice-cold buffer containing 50 mM Tris (pH 7.5), 150 mM NaCl, 5 % glycerol, 0.5 % Triton X-100, 5 mM EDTA, 50 mM NaF, 1 mM Na_3_VO_4_, 0.5 mM PMSF and supplemented with the complete protease inhibitor cocktail and used for the analysis. To remove proteins non-specifically binding to Glutathione Sepharose 4B or to glutathione S-transferase, the samples were pre-cleared by incubation with GST-bound Glutathione Sepharose 4B beads for 2 h at 4 °C, and the beads were removed by centrifugation at 18,000×*g* for 10 min at 4 °C. Approximately 10 μg of GST-MVI tail or GST alone were bound to 40 μl Glutathione Sepharose 4B beads per sample and then incubated for 4 h at 4 °C with equal amounts of the pre-cleared homogenate. The beads were exhaustively washed in the ice-cold buffer described above, and then boiled in SDS-loading buffer.

### Sample preparation and protein identification by LC-MS/MS

The samples obtained in the pull-down experiments were separated on SDS-PAGE in 10 % Tris–HCl precast gels and visualized by Coomassie R-250 staining as described by Majewski et al. (2012). After the electrophoretic separation, equal pieces were cut from the experimental (GST-fusion MVI globular tail) and control (GST alone) gel lanes. Prior to the analysis excised gel slices were subjected to the standard procedure of in-gel trypsin digestion, during which proteins were reduced with 100 mM DTT for 30 min at 56 °C, alkylated with iodoacetamide in darkness for 45 min at room temperature, and digested overnight with 10 ng/μl trypsin. Peptides were eluted from the gel with a water solution of 0.1 % FA and 2 % ACN and were applied to RP-18 pre-column (Waters, USA) using 0.1 % FA water solution as a mobile phase and then transferred to a nano-HPLC RP-18 column (internal diameter 75 μm, Waters, USA) using ACN gradient (0–30 % in 45 min) in the presence of 0.1 % FA at a flow rate of 250 nl/min. The column outlet was coupled directly to the ion source of LTQ FTICR mass spectrometer (Thermo Electron Corp., USA) working in the regime of data-dependent MS to MS/MS switch. A blank run ensuring absence of cross-contamination from previous samples preceded each analysis.

### Analysis of mass spectrometry data

After pre-processing the raw data with Mascot Distiller software (version 2.1.1, Matrix Science, London, UK), the obtained peak lists were used to search the non-redundant protein database of the National Centre for Biotechnology Information (NCBI) version 20090922 (9738651 sequences, 68328 rat sequences) using the Mascot search engine (version 2.2.03, 8-processors onsite license; Matrix Science, London, UK) with the following search parameters: taxonomy restriction—*Rattus norvegicus* (rat), enzyme specificity—semi-trypsin, permitted number of missed cleavages-1, fixed modification—carbamidomethylation (C), variable modifications—carbamidomethylation (K) and oxidation (M), protein mass—unrestricted, peptide mass tolerance, ±40 ppm, fragment mass tolerance, ±0.8 Da, max missed cleavages-1. Only proteins detected in two independent experiments and for which at least eight peptides with Mascot cut-off scores ≥96, indicating identity or extensive homology of a peptide (*p* ≤ 0.05) were identified in the GST-MVI tail sample and not in the GST alone sample, were considered as the positive identifications. MVI peptides detected in the sample were excluded from the analysis.

## Results

### Myosin VI expression in skeletal muscle

Western blot and RT-PCR techniques were used to detect myosin VI (MVI) in rat hindlimb muscles (soleus, EDL, and GM) and to assess its expression levels as well as its isoform pattern.

**Fig. 1:**
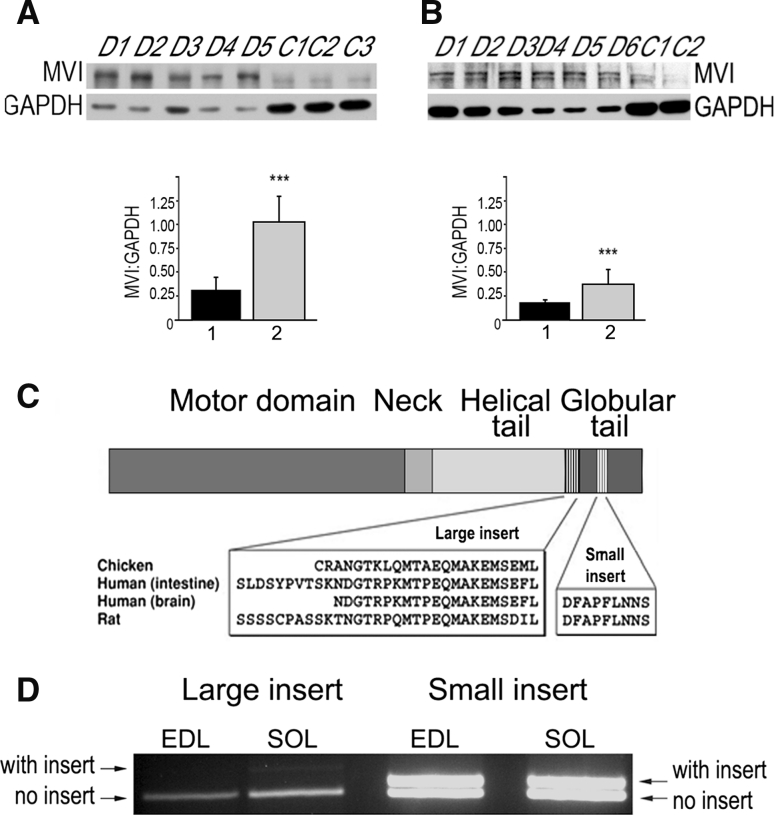
MVI expression in skeletal muscle. **a**, **b** Detection of MVI and GAPDH in homogenates of soleus and EDL muscles, respectively. Lanes marked with *C*, samples of control, untreated muscles, and with *D*, samples of denervated muscles. *Panels* below, densitometric analysis of bands detected with anti-porcine MVI and anti-GAPDH antibodies, where 1 and 2 are the means (±SD) for control and denervated muscles, respectively. The amount of MVI in tested samples is presented as MVI:GAPDH ratio. ***Statistical relevance measured with *t* Student’s test *p* < 0.001. **c** The diagram representing the domain organization of the MVI heavy chain. The sequence of large and small inserts is depicted as indicated, based on Au et al. (2007). **d** Assessment of MVI splice variants by RT-PCR in EDL and soleus (SOL) muscles. The products obtained with primers designed to produce fragments containing either small or large inserts, as indicated in the figure. Further details as described in “Materials and methods”

MVI was detected in rat hindlimb muscles, both intact and denervated (Fig. 1a, b), although expression was significantly greater in denervated muscles (after the sciatic nerve cut; Fig. 1a, b). GADPH expression was decreased in denervated muscles in comparison with intact muscles, indicative of impaired muscle metabolism (Wei et al. 2012).

Theoretically, four MVI posttranscriptional splice variants could be formed in mammals due to the presence of two inserts (large and small) in its C-terminal globular tail domain (Au et al. 2007; Aschenbrenner et al. 2003; Fig. 1c). To assess, which is/are expressed in skeletal muscle, RT-PCR technique was employed. As shown in Fig. 1d, in soleus and EDL muscles, a band corresponding to variant(s) with a small insert was easily seen, while a band corresponding to a variant with a large insert was barely detected. These data indicate that variants without the large insert predominate in the skeletal muscle we examined.

### MVI distribution in muscle fibers

Localization of MVI was examined by immunofluorescence in transverse and longitudinal sections of control and denervated hindlimb muscles.

**Fig. 2:**
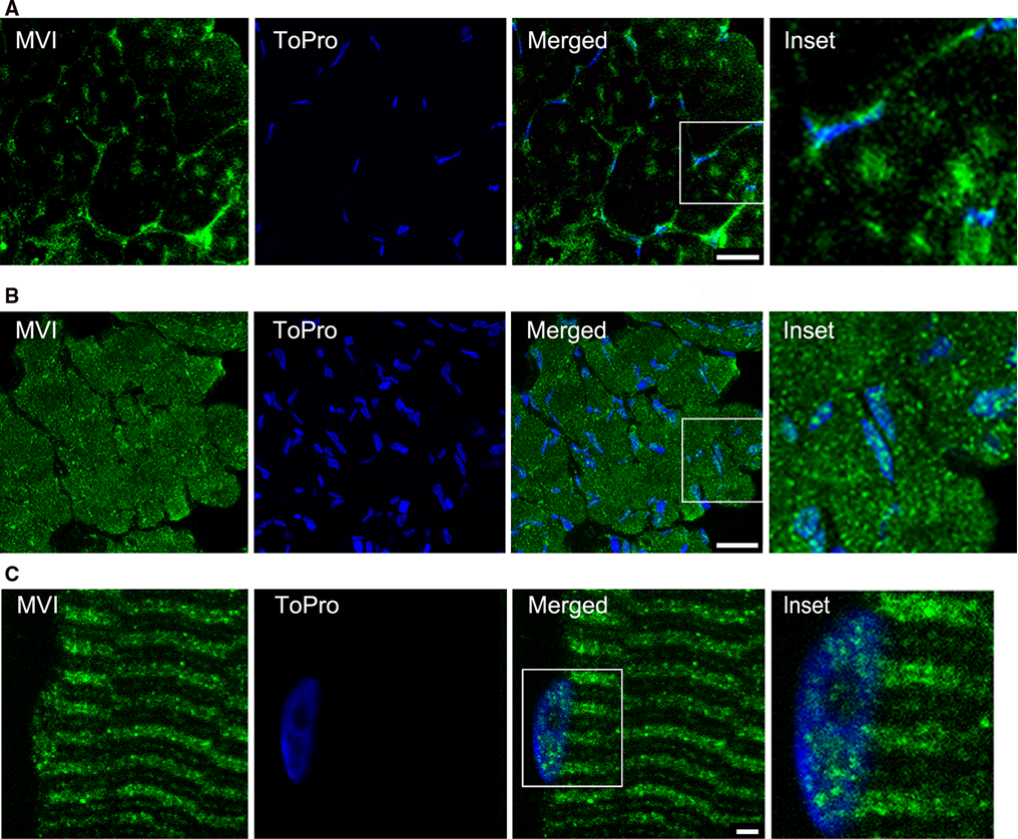
MVI distribution in the muscle fiber. **a**, **c** Transverse and longitudinal sections of control soleus muscle, **b** transverse section of 2-m denervated soleus muscle. The sections were stained with anti-MVI antibody (in *green*) and ToPro3 (*blue* marking nuclei). *Insets* about 2.5× magnification of the marked areas. These are 0.3 μm images of the fiber center obtained with a Leica confocal microscope. *Bars* 20 μm in **a**, **b**, and 5 μm in **c**

**Fig. 3:**
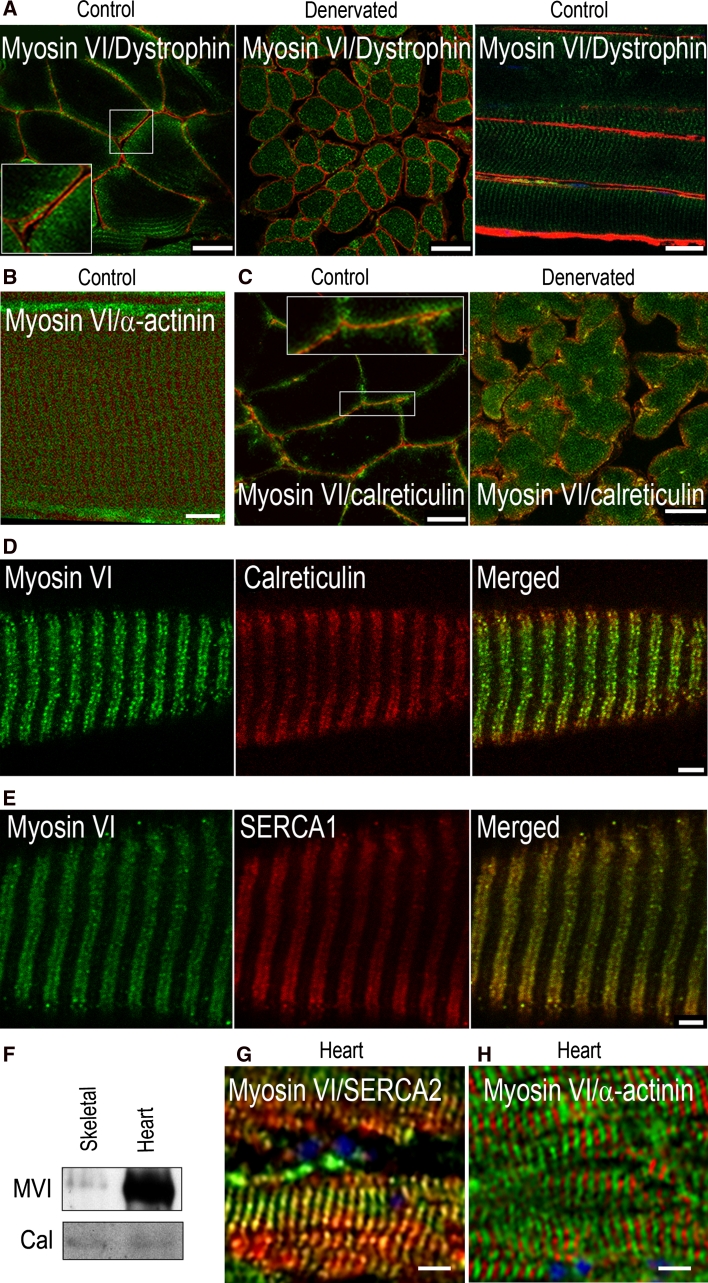
MVI is present in sarcoplasmic reticulum (SR). **a** Staining with anti-MVI (*green*) and anti-dystrophin (*red*) antibodies of transverse and longitudinal sections of control and 2-m denervated soleus muscle. **b** Staining with anti-MVI (*green*) and anti-α-actinin (*red*) antibodies of transverse sections of control EDL muscle. **c** Staining with anti-MVI (*green*) and anti-calreticulin (*red*) antibodies of transverse sections of control and 2-m denervated soleus muscle. *Insets* in **a**, **c**, about 2× magnification of the marked areas. **d** Staining with anti-MVI (*green*) and anti-calreticulin (*red*) antibodies of longitudinal section of control EDL muscle. **e** Staining with anti-MVI (*green*) and anti-SERCA1 (*red*) antibodies of longitudinal section of control EDL muscles. **f** Detection of MVI and calreticulin (Cal) in sarcoplasmic reticulum from the rat skeletal and cardiac muscles by means of western blot analysis. **g** Staining with anti-MVI (*green*) and anti-SERCA2 (*red*) antibodies of longitudinal section of murine cardiac muscle. **h** Staining with anti-MVI (*green*) and anti-α-actinin (*red*) antibodies of longitudinal section of murine cardiac muscle. In *blue*, ToPro staining for the nuclei. *Bars* 20 μm in **a**, **c**, 10 μm in **b**, **g** and **h**, and 3 μm in **d** and **e**. Other details as in the legend to Fig. 2

As shown in Fig. 2a, in the control soleus muscle MVI was localized predominantly in fiber peripheries and punctate structures across the fiber, it was also present in some of the muscle nuclei (Fig. 3a). In denervated soleus muscle (Fig. 2b), staining for MVI was much more intensive throughout the entire fiber. Higher MVI staining intensity was also observed within the nuclei (Fig. 2b). Increased MVI content was also observed in denervated soleus muscle homogenate using western blot (Fig. 1b). Longitudinal section of the soleus fiber confirmed the nuclear presence of MVI, and revealed ~3 μm-striation pattern of MVI distribution (Fig. 2c). The pattern of distribution of MVI in both EDL and GM muscles was almost identical to that observed in the soleus muscle.

Double staining for MVI and the sarcolemma marker, dystrophin, have confirmed that in muscles from control animals MVI is indeed present at the periphery of muscle fibers and not in the endomysium. Despite its peripheral distribution, MVI does not evidently co-localize with dystrophin, in both control and denervated soleus muscle (Fig. 3a). Also, no colocalization with α-actinin, the marker of Z disks, was observed (Fig. 3b). However, MVI co-localized with markers of the sarcoplasmic reticulum and T-tubules: calreticulin (Fig. 3c, d) and SERCA1 pump (Fig. 3e). The value of Pearson coefficient for both immunostaining exceeded 0.5, indicative of substantial colocalization.

Western blots revealed the presence of MVI in the sarcoplasmic reticulum fractions isolated from rat skeletal muscle and hearts (Fig. 3f). Notably, the amount of MVI detected in heart tissue was profoundly higher in comparison with skeletal samples. Analysis of longitudinal sections of murine hearts revealed that MVI was present in the sarcoplasmic reticulum but not in the contractile apparatus of cardiac muscle (Fig. 3g, h, respectively), similar to skeletal muscles. These observations, along with the report of Mohiddin et al. (2004) on the association of MVI gene mutation with cardiac hypertrophy, indicate that MVI could play an important role(s) in heart function.

Partial co-localization of MVI with *cis* Golgi network (GM130) and mitochondria (cytochrome *c* oxidase subunit I) markers was also observed (not shown).

The changes in MVI expression level and its distribution in the denervated and control hindlimb muscles suggest the presence of innervation-dependent factor(s) that could determine its synthesis and localization within the muscle fiber.

### MVI at the neuromuscular junction

The observation that MVI distribution within the fiber is associated with the state of muscle innervation prompted us to check whether MVI was present at the neuromuscular junction.

**Fig. 4:**
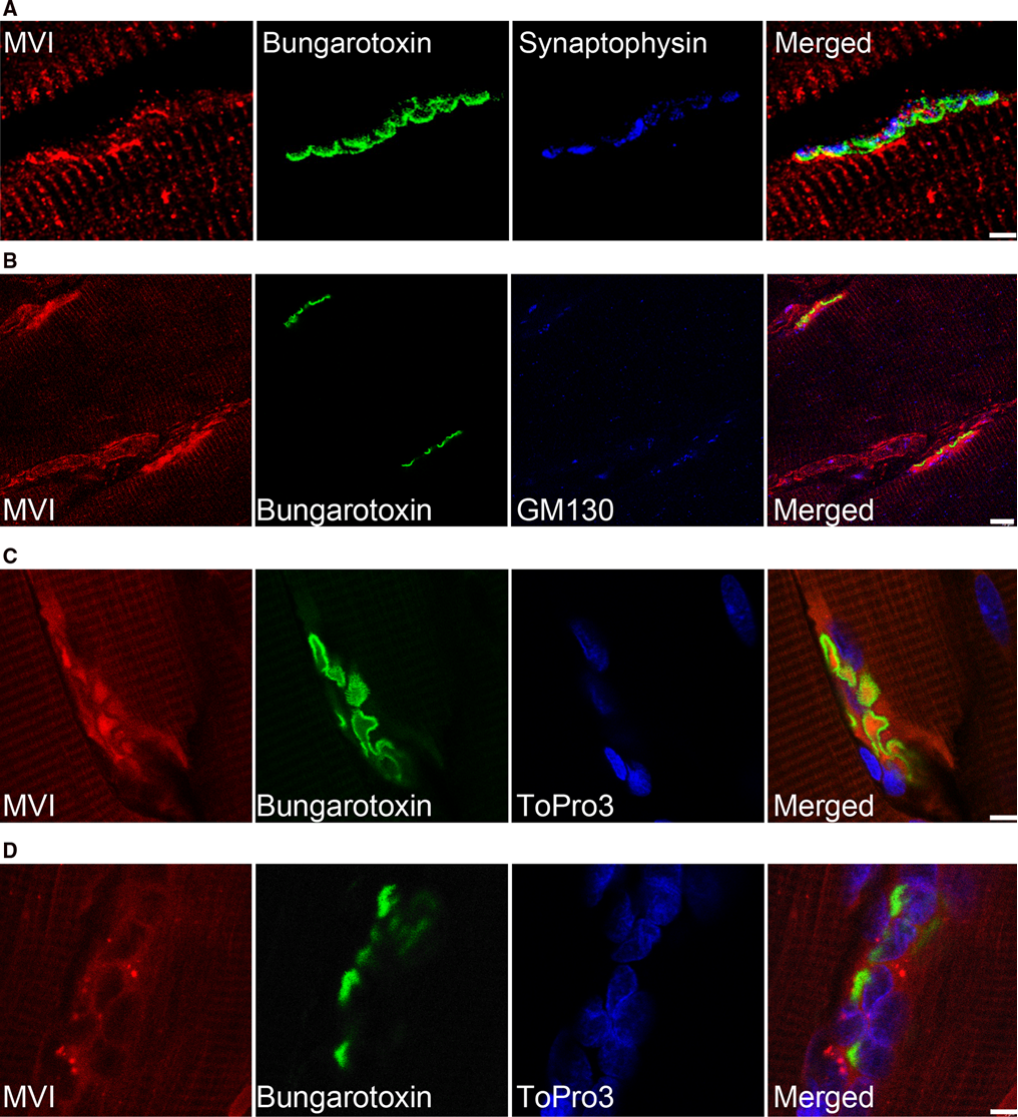
MVI in the neuromuscular junction. **a**, **b** Stainings of longitudinal sections of control gastrocnemius medialis (*GM*) muscle with anti-MVI antibody (*red*) and α-bungarotoxin (*green*), and in blue, stainings with the antibodies against synaptophysin (in **a**) and against GM130 (in **b**). **c**, **d** Stainings with anti-MVI antibody (*red*), α-bungarotoxin (*green*), and ToPro3 (*blue*) of longitudinal muscle section of EDL muscles from control (**c**) and ALS (**d**) rats. *Bars* 5 μm in **a**, **c** and **d**, and 10 μm in **b**. Other details as in the legend to Fig. 2

As shown in Fig. 4a–c, in control GM and EDL muscles, MVI accumulated within the areas stained with α-bungarotoxin (acetylcholine receptor blocking snake neurotoxin) and synaptophysin (marker of the synaptic vesicles). Analysis of the MVI staining (in the region behind the synapse, on the fiber side) indicates that MVI is located in the postsynaptic region of the junction (Fig. 4a). In the GM fiber region adjacent to the junction, MVI partially co-localizes with *trans* Golgi cisternae that are enriched in this area (Fig. 4b). Notably, MVI was absent in the nuclei of neuronal cells adjacent to the EDL muscle synapse, contrary to muscle nuclei (Fig. 4c).

To confirm whether MVI localization in the synapse is associated with the muscle innervation, the muscles isolated from the transgenic rats bearing a mutated superoxide dismutase gene (SOD^G93A^)—a model for ALS—were analyzed. As seen in Fig. 4d, MVI did not concentrate at the junction region even though the structure of the neuromuscular synapse was still preserved in the ALS tissue, as reported by Suzuki et al. (2008). This observation seems to suggest that MVI is rather not involved in neuromuscular junction integrity but could be engaged in transport of particles and vesicles at the muscle side of the junction.

### Identification of MVI-binding partners in the skeletal muscle

**Fig. 5:**
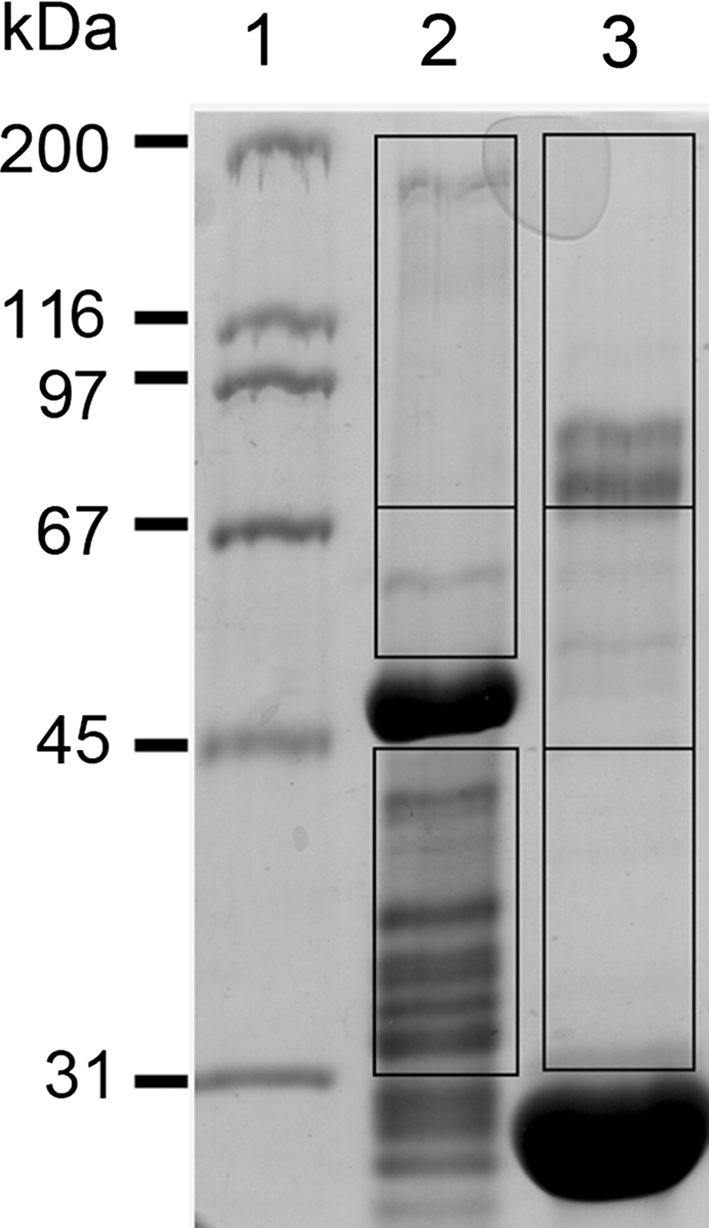
A search for MVI partners in skeletal muscles. Proteins of homogenates obtained from EDL muscle, which were bound either to MVI globular tail domain fused with GST (*lane 2*) or to GST alone (*lane 3*) were subjected to SDS gel electrophoresis.* Lane 1*, molecular weight standards. Gel pieces (marked on the figure) were excised and proceeded for mass spec analysis as described in “Materials and methods”. The proteins identified in the* lane 1* but not in the* lane 2* and fulfilled other criteria listed in “Materials and Methods” have been listed in Table 1

To identify MVI-interacting proteins in skeletal muscle, we performed affinity chromatography of the EDL muscle homogenate on GST-fused MVI globular tail domain (GST-GT-MVI, a cargo domain, see Fig. 1c) bound to glutathione Sepharose with subsequent detection of the bound proteins by tandem mass spectrometry. The lysates were pre-cleared by incubation with GST-bound beads prior to affinity chromatography, thus, the vast majority of unspecific interactions should be eliminated. Muscle homogenate proteins associated with the GST-GT-MVI (and in the control experiment with the GST alone) were subjected to SDS-PAGE electrophoresis and the gel pieces covering the entire lane were excised and subjected to mass spectrometry (Fig. 5). Only the proteins that met our criteria (i.e., were identified in two independent experiments, were present only in the GST-GT-MVI but not in control sample, and were identified by at least eight distinct peptide spectra) were considered as the potential MVI-binding partners (Table 1). It should be emphasized that with this method one cannot discriminate whether the identified proteins bind directly or indirectly to the MVI cargo domain.

**Table 1:** List of potential MVI binding partners in muscle homogenates identified by mass spectrometry

GI	Protein name	Experiment	n.p.	Score	%	Protein function
gi|56605806	Target of myb1 protein 1 (TOM1)	1	1	100	3	Intracellular trafficking; recruiting clathrin onto endosomes
		2	8	282	22	
gi|58865930	Fragile X mental retardation, autosomal homolog 1 (FMRP)	1	2	120	9	Transport of mRNA
		2	6	243	18	
gi|13384620	Heterogeneous nuclear ribonucleoprotein K (hnRNP K)	1	4	211	15	Transport and maturation of mRNA
		2	7	331	25	
gi|197927211	Heterogeneous nuclear ribonucleoprotein L isoform a (hnRNP L)	1	1	139	4	
		2	7	243	25	
gi|62078879	Family with sequence similarity 98, member A	1	3	178	9	unknown
		2	7	333	17	
gi|21703842	Hypothetical protein LOC28088	1	4	96	8	unknown
		2	15	545	31	

EDL muscle homogenate was subjected to GST affinity chromatography with rat MVI globular tail fused with GST attached to the glutathione Sepharose resin. After extensive washes, the beads were subjected to SDS gel electrophoresis (Fig. 5) followed by mass spectrometry analysis. Other details in “Materials and methods”. GI, protein ID in GenBank database; score, Mascot score;  %, percent of a protein polypeptide sequence matched by the identified peptides; n.p., a number of identified peptides for a given protein in the course of mass spec analysis

Out of 100 identified proteins only 6 met our criteria, among them were the target of myb1 protein 1 (TOM1), a protein involved in intracellular trafficking by recruiting clathrin onto endosomes (Katoh et al. 2004), as well as fragile X mental retardation protein (FMRP), which is involved in mRNA transport and in cardiac muscle development and functioning (Khandjian et al. 1998; Whitman et al. 2011). Other proteins involved in transport and maturation of mRNA, heterogeneous nuclear ribonucleoproteins K and L have been identified as well. Additionally, a member of a family with sequence similarity 98 and hypothetical protein LOC28088 with unknown functions have been identified.

## Discussion

In this study, we have addressed for the first time the question concerning the putative role of MVI in striated muscle. MVI was detected in fibers of rat hindlimb muscles, where it localized predominantly to the sarcoplasmic reticulum (also in cardiac muscle), muscle nuclei and the postsynaptic region of the neuromuscular junction. This well-defined MVI localization was impaired in the muscle fibers of animals subjected to a sciatic nerve cut (2-month denervated muscles) as well as in muscle nerve termini obtained from a rat model of ALS.

### Distribution and expression of MVI in muscle fibers

We did not find any difference in the distribution and amount of MVI, and its splice variants, between slow and fast muscle of control rats. Only a few unconventional myosins have been found in myogenic cells and/or striated muscle so far (Wells et al. 1997; Redowicz 2007). The expression levels of most of them decrease during myogenesis, for instance of myosins IB and IE, VA, VIIB and IXB (Wells et al. 1997).

The predominant presence of MVI variants without a large insert is characteristic for non-polarized cells (Au et al. 2007; Aschenbrenner et al. 2003). Interestingly, the amount of MVI has been greatly enhanced in denervated muscles. Also, we observed that MVI expression was increased in the atrophic fibers of muscle biopsies obtained from patients with myopathies (J. Karolczak, unpublished observation). These observations suggest the importance of this molecular motor in the complex processes associated with atrophy and fiber transformation, which take place in denervated muscle (Jakubiec-Puka et al. 2008).

### MVI in the sarcoplasmic reticulum

In the skeletal and cardiac muscles, MVI localized mainly to markers of the sarcoplasmic reticulum (SR), one of the major membranous muscle compartments, which is specialized in calcium homeostasis and control of muscle contraction (Sorrentino 2011). This is in line with our earlier observation that MVI localizes to the endoplasmic reticulum of neurosecretory PC12 cells (Majewski et al. 2010). Moreover, we detected MVI in the SR fractions isolated from both rat fast twitch muscle and cardiac ventricular muscle. Not a single SR-specific protein was detected in the pull-down sample subjected to the mass spectrometry analysis. We believe that this could be due to methodological limitations, both during the pull-down chromatography or the sample proceeding during mass spectrometry, but not lack of the interaction(s) of MVI with the SR component(s). We presume that in the SR MVI could be involved in transport of various particles within this giant muscle structure or in maintenance of this organelle. The involvement of MVI in the vesicle and particle trafficking within the SR (and other muscle compartments) seems to be confirmed by identification in our mass spectrometry analysis of the target of mybp1 (TOM1) protein, which participates in intracellular trafficking by recruiting clathrin and ubiquitin-conjugated proteins onto endosomes (Katoh et al. 2004; Yamakami et al. 2003). Interestingly, its Drosophila ortholog (CG3529) was identified in a proteomics approach aimed at understanding functioning of MVI in flies (Finan et al. 2011). Moreover, it has been recently shown that the interaction between MVI and TOM1 is involved in autophagosome maturation (Tumbarello et al. 2012). No other known MVI partner involved in endocytosis, such as GIPC or Dab2, was identified in our sample. Also, in another study on muscle biopsy from a patient with centronuclear myopathy, caused by a mutation within the dynamin 2 gene, we detected MVI on the surface of the calthrin-, muscle synapse component, and dystrophin-containing vacuoles accumulated within the fibers, which further confirms a role of MVI in vesicular trafficking within muscle fibers (Karolczak et al. 2011). It was shown that MVI via interaction with optineurin is involved in the maintenance of the Golgi cisternae (Sahlender et al. 2005) thus it is plausible that it could play similar role(s) in the SR maintenance.

MVI binds two calmodulins per its heavy chain, therefore, it could theoretically participate in calcium homeostasis. However, direct involvement of MVI in this complex phenomenon is rather unlikely since calmodulin and calcium ions are involved in the regulation of the intrinsic MVI activity (Prochniewicz et al. 2011). On the other hand, MVI activation leads to interaction with filaments formed by non-muscle actin (β and γ) isoforms, which are the building blocks of the non-sarcomeric cytoskeleton (present also within SR) and thus, MVI-evoked calcium-dependent reorganization of the cytoskeleton architecture could affect functions of many of the SR components (Kee et al. 2009).

### MVI in the neuromuscular junction

We have also detected MVI in the postsynaptic region of the neuromuscular junction indicating its possible involvement in neuromuscular transmission. The presence of non-muscle myosins IIA and IIB as well as unconventional myosin VA in the muscle synapse has been already reported (Vega-Riveroli et al. 2005; Roder et al. 2008). Myosins IIA and IIB have been found in the adult motor nerve terminals and may play a role in the maintenance of plasticity at the neuromuscular junction (Vega-Riveroli et al. 2005). The important role of myosin VA in the plasticity of the vertebrate junction in vivo has been demonstrated since lack of myosin VA caused severe fragmentation and size reduction of the muscle synapse, and reduced persistence of acetylcholine receptors in the junction (Roder et al. 2008). Studies on Drosophila MVI gene (*jaguar*) have revealed its contribution in synaptic transmission and development (Kisiel et al. 2011). Our observation on the postsynaptic localization of MVI is in agreement with the data obtained from hippocampal neurons where MVI was found on the post-synaptic densities and shown to play important roles in the synapse and dendritic spine formation, internalization of glutamate receptors and BDNF-mediated neurotransmission as well as in localization of axonal proteins (Yano et al. 2006; Osterweil et al. 2005; Lewis et al. 2011). Our study indicates that in adult muscles MVI could be involved in neuromuscular transmission (probably by transporting particles and vesicles at the fiber side of the muscle synapse) and not in junction integrity since its postsynaptic presence was significantly reduced in the ALS rat muscle junction, where its architecture was still preserved even though neuronal transmission was most probably impaired (Suzuki et al. 2008).

### MVI in the muscle nuclei

The presence of MVI within the nucleus has been already reported for many cancer cell lines, including neurosecretory pheochromocytoma PC12 cells, prostate cancer and HeLa cells (Vreugde et al. 2006; Jung et al. 2006; Majewski et al. 2010). Interestingly, non-muscle actin and at least four other unconventional myosins have been found within the nucleus, where they play important roles in transcription and nuclear transport (de Lanerolle and Serebryannyy 2011). The data gathered so far indicate that MVI is involved in RNA polymerase II-dependent transcription and nascent transcript maturation (Vreugde et al. 2006; Jung et al. 2006; Majewski et al. unpublished observation). Interestingly, the presence of MVI in the nuclei of denervated hindlimb muscle was greatly enhanced, as was the total amount of MVI within the entire fiber. Also, the increased level of MVI was detected in the centrally positioned nuclei of a patient with central nuclear myopathy (Karolczak et al. 2011). This enhanced nuclear localization could be a compensatory response driven by the increased need for this motor protein in the diseased muscle. The complex phenomena taking place in denervated muscles, which are associated with actively occurring fiber degeneration and its simultaneous recovery require not only increased particle trafficking within the fiber, but also enhanced transcription to synthesize new proteins to rebuild damaged fiber components. Notably, we have not detected MVI in the nuclei of the Schwann cells adjacent to the neuromuscular junction thus indicating that its nuclear presence may be specific for muscle cells. It is plausible that MVI could be involved in transcription of muscle-specific genes, as has already been proposed for myosin XVIIIB (Salamon et al. 2003; Ajima et al. 2008). Detection in the fraction of potential MVI-binding partners of FMRP and hnRNP proteins, which are known to be involved in transport and nascent transcript maturation seems to further confirm our suggestion on involvement of MVI in transcription and transcription-related processes. In line with our observation, the interaction of MVI with TLS, a protein involved in rapid nuclear-cytoplasmic shuttling of spliced mRNA, was previously shown in murine hippocampus (Tamaki et al. 2009).

### MVI-binding partners

It is generally accepted that MVI fulfills its functions through interactions with actin and a set of interaction partners bound to the MVI C-terminal globular tail domain (cargo domain) in the tissue- and/or cell type-specific manners. Our mass spectrometry analysis has identified several potential MVI partners, which are known to participate in different muscle functions. As mentioned above, among them are proteins involved in endocytic trafficking (TOM1) as well as in transport of mRNA (FMRP) and in nascent transcript maturation (hnRNPs). It should be underlined that FMRP was shown to repress translation mRNAs of desmoplakin and talin2, proteins involved in formation of desmosomes as well as costameres, the key structures for regulating the heart (Khandjian et al. 1998; Whitman et al. 2011). In line with this, in PC12 cells hnRNPs were also found among potential MVI partners, and hnRNP U colocalized with MVI in the nuclei of stimulated cells (Majewski et al., unpublished observation). Further studies are required to verify and characterize these novel interactions, and their regulatory mechanisms.

## Conclusions

We have addressed, for the first time, the role of MVI in striated muscles by characterization of its expression and splice variant formation in rat hindlimb muscle, its distribution in both normal and denervated skeletal muscles as well as in ventricular cardiac muscle, and by identification of proteins binding to the MVI cargo domain (potential MVI partners) in muscle homogenates. Our observations indicate that in striated muscles MVI could be involved in intra-fiber trafficking, in the SR and sarcomere maintenance, and possibly in neuromuscular signal transmission and in transcription. We believe that this study aids in understanding the role of MVI in striated muscle, and in explanation of the clinical phenotype associated with a point mutation (H246R) within MVI motor domain manifested not only with deafness (as is observed for all vertebrate MVI mutations), but also with cardiac hypertrophy (Mohiddin et al. 2004).

## Electronic supplementary material

Below is the link to the electronic supplementary material.

Supplementary material 1 (PDF 88 kb)
