# Supplementary material for: Myosin VI in skeletal muscle: its localization in the sarcoplasmic reticulum, neuromuscular junction and muscle nuclei
Source: Histochem Cell Biol. 2012 Dec 30;139(6):873–85. doi: 10.1007/s00418-012-1070-9 (PMC3656228; doi:10.1007/s00418-012-1070-9)
Supplement: Supplementary file 1 — Supplementary material 1 (PDF 88 kb) [file 418_2012_1070_MOESM1_ESM.pdf]

Title: Myosin VI in skeletal muscle: Its localization in the sarcoplasmic reticulum, neuromuscular junction and muscle nuclei

Journal: Histochemistry and Cell Biology

Justyna Karolczak, Magdalena Sobczak, Łukasz Majewski, Marine Yeghiazaryan, Anna Jakubiec-Puka, Elisabeth Ehler, Urszula Sławińska, Grzegorz M. Wilczyński and Maria Jolanta Rędowicz*

* Corresponding author:
Maria Jolanta Rędowicz
Nencki Institute of Experimental Biology
Department of Biochemistry; 3 Pasteur St., 02-093 Warsaw, Poland
e-mail: j.redowicz@nencki.gov.pl; Tel. +48-22-5892456; Fax: +48-22-8225342

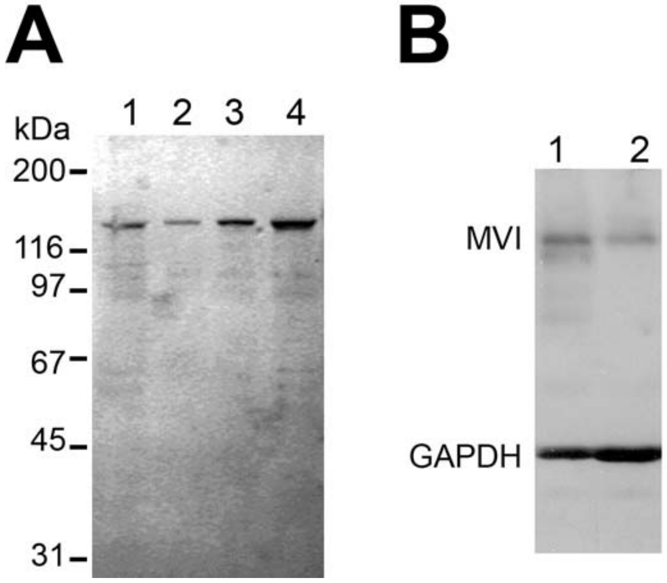

Online resource 1. Detection of myosin VI heavy chain in muscle homogenates. **A.** Myosin VI heavy chain (M.W. ~140 kDa) was detected with polyclonal anti-porcine myosin VI antibody in homogenates of mouse brain (lane 1, 10 μg) and rat soleus muscle (lanes 2-4). Lanes 2-4, 10, 20 and 40 μg of the homogenate, respectively. Myosin VI band was visualized using alkaline phosphatase-based detection system. **B**. Myosin VI heavy chain and GAPDH (glyceraldehyde 3-phosphodehydrogenase) were detected in homogenates of rat soleus (lane 1) and EDL (lane 2 muscles). ~10 μg of homogenates were loaded onto the gel. The band corresponding to the proteins were visualized using the enhanced chemiluminescence (ECL) detection system.
